# Epidemiology of Enterotoxigenic *Escherichia coli* among Children and Adults Seeking Care at Hospitals in Two Geographically Distinct Rural Areas in Bangladesh

**DOI:** 10.3390/microorganisms12020359

**Published:** 2024-02-09

**Authors:** Subhra Chakraborty, Fatema-Tuz Johura, Marzia Sultana, Xueyan Zhang, Abdus Sadique, Christine M. George, Shirajum Monira, David A. Sack, Richard Bradley Sack, Munirul Alam

**Affiliations:** 1 Johns Hopkins Bloomberg School of Public Health, Johns Hopkins University, Baltimore, MD 21205, USA; mjohura1@jh.edu (F.-T.J.); xzhan211@alumni.jh.edu (X.Z.); cgeorge19@jhu.edu (C.M.G.); dsack1@jhu.edu (D.A.S.); rsack@jhsph.edu (R.B.S.); 2 International Centre for Diarrhoeal Disease Research, Dhaka 1212, Bangladesh; msultana@icddrb.org (M.S.); sadique004@gmail.com (A.S.); smonira@icddrb.org (S.M.); munirul@icddrb.org (M.A.)

**Keywords:** Enterotoxigenic *E. coli*, diarrhea, epidemiology, colonization factors, subnational, seasonal assessments, Bangladesh, adults, children

## Abstract

Enterotoxigenic *Escherichia coli* (ETEC) infections undeniably continue to have substantial morbidity and mortality in younger children; however, limited data are available on the disease burden of older children and adults and on ETEC epidemiology by geographical location at the subnational level. Facility-based surveillance over the years was established to identify patients with ETEC diarrhea in two geographically distinct areas in rural Bangladesh, Chhatak in the north and Mathbaria in the southern coastal area. ETEC was highly prevalent in both areas, while the proportions, toxin types and colonization factors varied by location, season and age groups. Children < 5 years old and adults between 20 and 60 years old were at the highest risk of ETEC diarrhea which required urgent care. This study underscores the importance of capturing subnational and seasonal variations in ETEC epidemiology. ETEC vaccine developers and public health stakeholders may need to target adults between 20 and 60 years of age in addition to young children as new vaccines currently under development become licensed and introduction begins.

## 1. Introduction

Enterotoxigenic *Escherichia coli* (ETEC) is the most common bacterial cause of diarrhea in children in low- and middle-income (LMIC) countries [1,2,3,4]. ETEC is also the most common cause of traveler’s diarrhea (TD), responsible for 30 to 60% of all TD cases [5,6]. Besides causing high rates of morbidity and mortality, expanded effects of non-fatal ETEC diarrhea and subclinical infections can have long-term consequences on the development of children, considerably exacerbating the impact of these infections [7]. Moreover, the widespread use of antibiotics contributes to the increased spread of antimicrobial-resistant strains of ETEC and *E. coli*. Although there are many candidate ETEC vaccines under development, including the most advanced ETVAX vaccine [8,9], none are currently licensed. 

Data on the burden of ETEC are not readily available from LMICs, which has created uncertainties in the reported morbidity and mortality estimates. The lack of data could be attributed to the complex laboratory methods required to detect ETEC, which are not feasible outside of specialized laboratories [3,10,11]. It is estimated that ETEC causes about 220 million diarrhea episodes globally, with about 75 million episodes in children under 5 years of age, resulting in between 18,700 deaths [Institute for Health Metrics and Evaluation (IHME) estimates] and 42,000 deaths [Maternal and Child Epidemiology Estimation Group (MCEE) estimates] among children younger than 5 years [3,9,12,13]. 

The efforts to control ETEC diarrhea are primarily focused on children under five years old. Limited data are available on the ETEC burden among older children and adults from LMICs. A meta-analysis projected that ETEC might contribute to an additional 89,000 deaths per year among age groups older than five years in Africa and South Asia [14]. Further studies are needed to determine the risk and disease burden beyond 5 years old to determine if interventions to control ETEC should also target this population. 

ETEC is endemic and a major cause of diarrhea in Bangladesh [15,16,17]. However, epidemiological studies of ETEC in Bangladesh have primarily been conducted in urban Dhaka and its suburbs, located in central Bangladesh, and data are limited from the rural areas. Studies of infectious disease incidences have shown the importance of geographical differences and variable disease risk and burden at the subnational level when determining the impact and cost-effectiveness of intervention strategies [3,7,9,12,13,18]. 

In this report, we describe the findings from the health-facility-based surveillance of ETEC conducted over the years in the two geographically distinct rural sentinel sites, Chhatak in the north and Mathbaria in the south of Bangladesh. We analyzed the seasonal occurrence and epidemiology of ETEC among all ages in these areas and determined the associations between toxin types, colonization factors (CFs) of ETEC and the severity of diarrhea in patients. Identifying the population at risk and understanding the geographical variations in ETEC epidemiology within a country would help design and target ETEC vaccines and other interventions to the most vulnerable population.

## 2. Materials and Methods

This is an observational, hospital-based surveillance study of patients presenting acute watery diarrhea at the two health facilities in Bangladesh. This ETEC study was conducted using the centennial surveillance established by the “Epidemiology and ecology of cholera” study [19]. 

### 2.1. Surveillance

**Figure 1:**
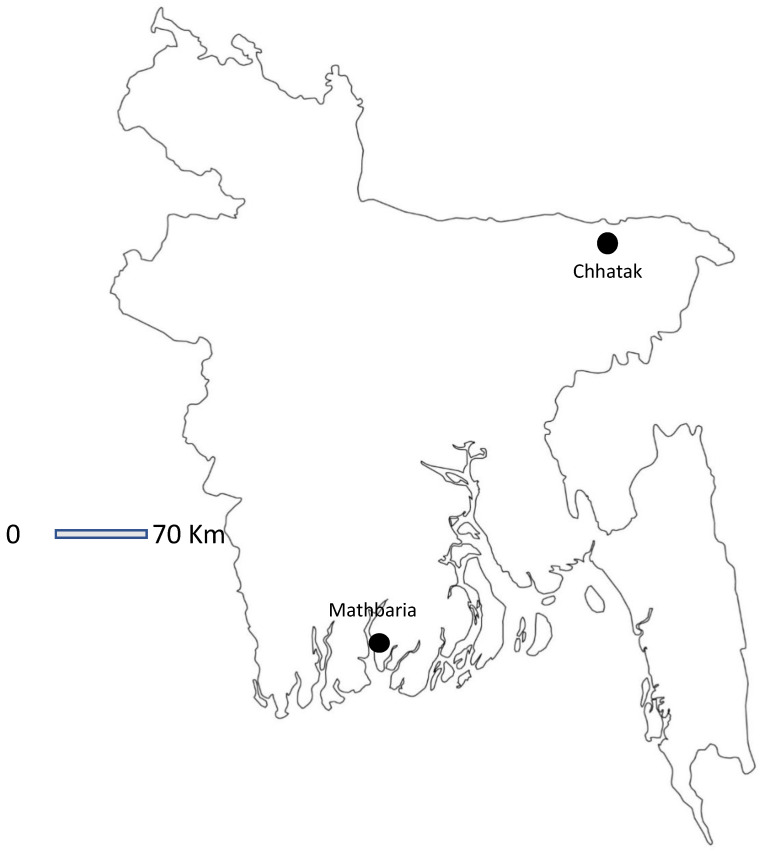
Map of Bangladesh showing areas where the epidemiological surveillance of ETEC was conducted.

The ETEC surveillance study was conducted from 2014 to 2015 in Chhatak and 2014 to 2016 in Mathbaria. Chhatak is situated in the flood plains of the river Brahmaputra in the north of the country in the Sunamganj district, 40 km from the divisional town of Sylhet (Figure 1). Mathbaria in Barishal Division is in the coastal southern part of the country, where the ground water is salty (Figure 1). This area is likely a representation of the rural villages along the ~575 km long coastline in Bangladesh. These clinical surveillance sites were chosen for several reasons. These two areas are far apart (~500 km) and geographically distinct. Each site has a health facility with a ward dedicated for treatment of diarrhea patients which is run by the Ministry of Health of the Government of Bangladesh. Travel was possible to each site throughout the year. Each facility had staff willing to participate in the clinical surveillance. Lastly, each site had a catchment area of roughly 150,000–200,000 people, although individuals living closer to the facility were expected to be more likely to attend than ones living at a distance [19].

The surveillance occurred for 3 days per week during “high” seasons, e.g., seasons when higher rates of diarrhea were expected based on previous years’ patterns, and for 3 consecutive days per month during “low” seasons, e.g., seasons when few cases of diarrhea were expected. This strategy was adopted with more sampling days during the high seasons, to allow for an increased number of cases to be detected and documented. A questionnaire was administered regarding clinical outcomes, demography and water sources for drinking, bathing and washing. 

After obtaining informed consent from patients with acute watery diarrhea, rectal swabs (RSs) were collected during the initial phase of the study from both of the sites, and this was replaced by stool samples in the later years in Mathbaria. Rectal swabs were placed in Cary Blair media and the stool samples were kept cold until transported to the microbiology laboratory at the icddr,b in Dhaka within 72 h.

### 2.2. Detection of ETEC from Stool Samples 

Rectal swabs or stool samples were cultured on MacConkey agar. Five lactose-fermenting *E. coli*-like colonies were selected and tested using a conventional multiplex PCR targeting the toxin genes heat-labile toxin (LT) and heat-stable toxin (STh and STp). Template DNA was prepared from the whole colony by boiling in a water bath for 10 min and instantly cooling on ice. Conventional PCR amplification was performed as described before [11] (Supplementary Table S1). The reactions were performed using SimpliAmp Thermocycler (Applied Biosystems, Foster City, CA, USA). We also tested the antimicrobial resistance of the ETEC strains [20].

Quantitative PCR (qPCR) was performed as described previously [11] on all of the stool samples collected from the later years in Mathbaria. DNA was extracted from 200 to 300 mg of solid stool or up to 500 ul of loose stool using a bead beater to disrupt cells, followed by processing using the Qiagen QIAamp DNA stool-extraction kit (Qiagen, Hilden, Germany). qPCR was conducted with the isolated DNA to detect the target genes, LT, STh and STp, for ETEC using the Step One Plus Real-Time PCR System (Applied Biosystems, Foster City, CA, USA) with SYBR Green-based fluorescent dye [11]. Each sample was run in duplicate, and results were averaged. ETEC strain H10407 (O78:H11 LT^+^, STh^+^, STp^+^) and ATCC 25922 were used as the positive and negative controls, respectively, for PCR and qPCR. 

### 2.3. Detection of CFs 

Genomic DNA was tested for the presence of 14 CF genes CFAI, CS1-8, CS12, CS17, CS17/19, CS20 and CS21 using three separate panels of 5-plex PCRs [21] followed by confirmation with simplex conventional PCR. 

### 2.4. Serotyping

The ETEC-positive colonies were screened for O-antigen serogroups, as described before [22]. Colonies were cultured and slide agglutination was performed using O-antigen antisera (Denka Seiken, Tokyo, Japan), first with polyclonal followed by monoclonal antisera. 

### 2.5. Statistical Analysis

Comparisons were carried out where necessary using SigmaStat statistical software version 4.0. (Jandel scientific, San Rafael, CA, USA). R version 2.14.1 for Chi-square tests was used for significance analysis. For qPCR analysis, any samples with quantification cycles (Cq) of 30 or less for any one of the ETEC toxin genes, either LT, STh or STp, were considered ETEC-positive. Cq30 corresponded to ~10^5^ CFU/gm of stool [11]. 

### 2.6. Ethics Statement 

This study was approved by the institutional review boards of the icddr,b and Johns Hopkins University. Written informed consent was obtained from the adults and the caregivers of the children who participated in this study. 

## 3. Results

In Chhatak, from April 2014 to January 2015, stool samples were collected from 174 patients with diarrhea seeking care in the Upazila Health Complex. The stool samples were screened using culture followed by conventional PCR on isolated *E. coli*-like colonies, and 14.4% (25 of 174) were positive for ETEC (Table 1).

During March 2014 to December 2016, stool samples were collected from 354 patients seeking care in the Mathbaria Upazila Health Complex (Table 1). Due to local political reasons, there were disruptions in traveling to the site from January to February in 2015, which created a gap in sample collection. Overall, the isolation rate of ETEC in Mathbaria was 11.3% (40 of 354) using conventional PCR, with the highest (19.1%) in 2014. There were more females with ETEC diarrhea than males in Mathbaria compared to Chhatak (Table 1). 

Among the ETEC-positive diarrhea cases in Chhatak, 48% were ST-ETEC, followed by 40% LT-ETEC and 12% LT+ST-ETEC (Table 2). There were no STp-ETEC cases detected. In Mathbaria, ST-ETEC was the most prevalent, constituting 70.0% of the total ETEC cases, followed by LT+ST-ETEC at 22.5%, while only 7.5% of cases were positive for LT-ETEC (Table 2). There were five STp-ETEC cases detected from Mathbaria. 

In Mathbaria, 260 stool samples from March 2015 to June 2016 were reanalyzed with qPCR. The isolation rate of ETEC with qPCR was 23.8% (62 of 260), which was about 2-fold higher than that of multiplex conventional PCR performed from *E. coli* isolates. All the samples which were positive by conventional PCR for ETEC were also positive by qPCR. Although there was an increase in LT genes with qPCR, the proportion of the LT-ETEC still remained the lowest compared to ST-ETEC and LT+ST-ETEC.

### 3.1. The Risk of ETEC Diarrhea among Age Groups

**Figure 2:**
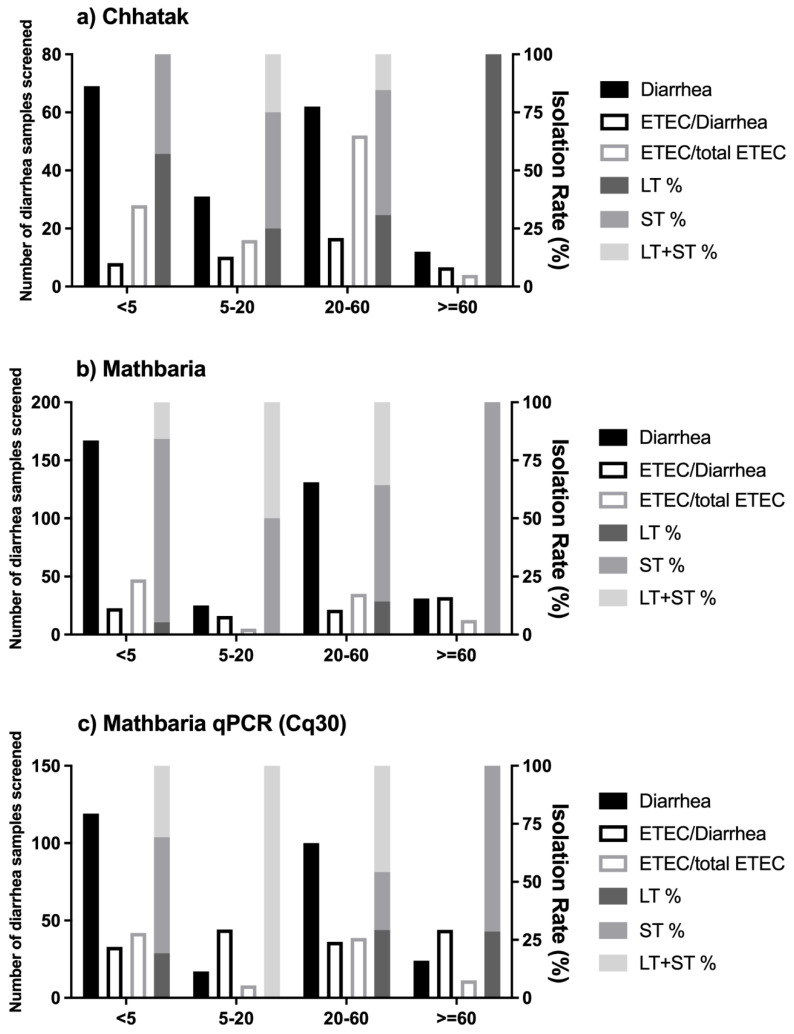
Isolation rates of ETEC and toxin types by age groups: (**a**) ETEC-positive diarrhea cases detected with conventional PCR in Chhatak; (**b**) ETEC-positive diarrhea cases detected with conventional PCR in Mathbaria; (**c**) ETEC-positive diarrhea cases detected with qPCR in Mathbaria. Black-filled bar: number of diarrhea samples screened, left y axis. Black-outlined bar: number of ETEC cases divided by number of diarrhea samples screened, right y axis. Grey-outlined bar: number of ETEC cases divided by total number of ETEC cases, right y axis. Dark-grey-filled bar: number of LT-ETEC cases divided by total number of ETEC cases, right y axis. Medium-grey-filled bar: number of ST-ETEC cases divided by total number of ETEC cases, right y axis. Light-grey-filled bar: number of LT+ ST-ETEC cases divided by total number of ETEC cases, right y axis.

Chhatak: Overall, among the 174 diarrhea patients enrolled in Chhatak, 39.7% (69) were under 5 years old, of whom 70% (48) were <2 years old. The isolation rate of ETEC among the diarrhea patients of <5 years was 10.1% (7 of 69); in those of ≥5 to 20 years, it was 12.9% (4 of 31); in those of ≥20 to 60 years, it was 21% (13 of 62); and in those of ≥60 years of age, it was 8.3% (1 of 12) (Figure 2a). 

Among the 25 ETEC diarrhea cases, 28% of patients were <5 years old, of whom 57.1% had LT-ETEC and 42.9% had ST-ETEC; 16% were 5 to 20 years old, of whom 50% had ST-ETEC and 25% each had LT-ETEC and LT+ST-ETEC; 52% were 20 to 60 years old, of whom 53.8% had ST-ETEC, followed by 30.8% LT-ETEC and 15.4% LT+ST-ETEC, and 1 patient (4%) who was positive for LT-ETEC was >60 years old (Figure 2a). 

Mathbaria: Overall, among the 354 diarrhea patients enrolled, 47.2% (167) were under 5 years old, of whom 80.8% (135) were <2 years old. The isolation rate of ETEC using conventional PCR among the diarrhea patients < 5 years old was 11.4% (19 of 167); in those ≥ 5 to 20 years old, it was 8% (2 of 25); in those ≥ 20 to 60 years old, it was 10.7% (14 of 131); and in those ≥60 years old, it was 16.1% (5 of 31) (Figure 2b). Using qPCR, 21.9% of the diarrhea cases among patients of <5 years, 29.4% among those of ≥5 to 20 years, 24% among those of ≥20 to 60 years and 29.2% among those of ≥60 years of age were positive for ETEC (Figure 2c).

Among the 40 ETEC-positive diarrhea cases, 47.5% of the patients were <5 years old, of whom 78.9% had ST-ETEC, 15.8% LT+ST-ETEC and 5.3% LT-ETEC; 5% were 5 to 20 years old, of whom 50% each had ST-ETEC and LT+ST-ETEC; 35% were 20 to 60 years old, of whom ST-ETEC accounted for 50%, followed by 35.7% LT+ST-ETEC and 14.3% LT-ETEC; 12.5% were patients >60 years old, and all of them were ST positives. Using qPCR, the proportions of ETEC types by age were similar to the conventional PCR, except among patients of ≥5 to 20 years, with an increase in the detection of LT, and all the ETEC positives were LT+ST-ETEC. 

### 3.2. CFs and O ETEC Serogroups in Chhatak and Mathbaria

**Figure 3:**
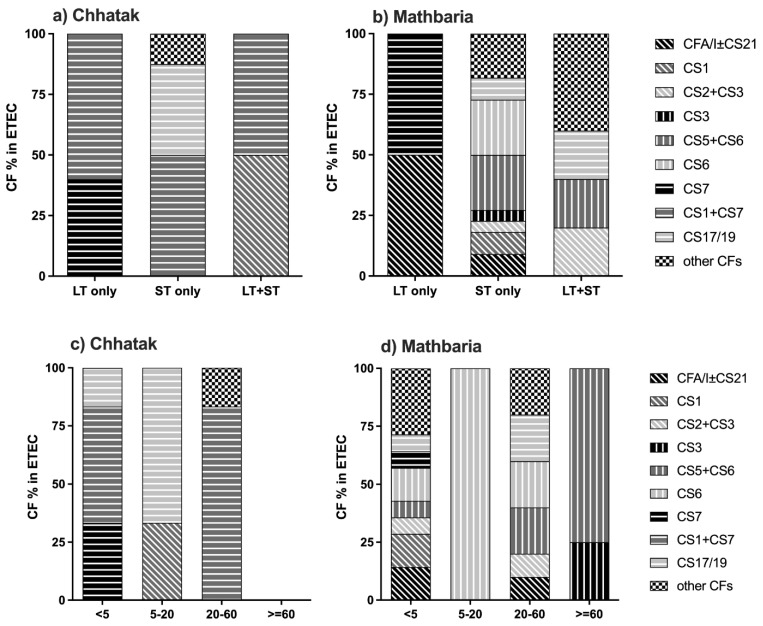
ETEC CFs by toxin distribution and by age group. (**a**) ETEC CFs by toxin in Chhatak. (**b**) ETEC CFs by toxin in Mathbaria. Data are shown as the number of ETEC cases with each CF divided by the number of ETEC cases with specific toxin(s) genes. (**c**) ETEC CFs by age group in Chhatak. (**d**) ETEC CFs by age group in Mathbaria. Data are shown as the number of ETEC cases with each CF divided by the number of ETEC cases from each age group.

ETEC isolates from 19 and 35 ETEC-positive patients were available from Chhatak and Mathbaria, respectively, for the testing of CFs. In Chhatak, 78.9% (15/19) of the ETEC isolates had detectable CFs. The major CFs were CS1+CS7 (53.3%), CS17/19 (20%), CS7 (13.3%) and CS1 (6.7%). CS1+CS7 were associated with all the toxin types of ETEC, CS7 with LT, CS17/19 with ST and CS1 with LT+ST only (Figure 3a and Supplementary Table S2). The major CFs that were detected in the ETEC isolates from patients <5 years old were CS1+CS7 (50%), CS7 (33.3%) and CS17/19 (16.7%); from 5 to 20 years, they were CS17/19 (66.7%) and CS1 (33.3%); from 20 to 60 years, they were CS1+CS7 (83.3%) (Figure 3c and Supplementary Table S3). CFs could not be detected in the ETEC strains isolated from the patients who were more than 60 years old. 

In Mathbaria, overall, 82.9% (29 of 35) of the ETEC isolates had detectable CFs, ranging from 66.7 to 88.2% during the study years. The major CFs were CS5+CS6 (20.7%), CS6 (17.2%), and CFA/I±CS21 and CS17/19 (10.3%). CS5+CS6 was associated with ST and LT+ST, CS6 with ST only, CFA/I±CS21 and CS17/19 mainly with ST and a few LT+ST cases, and CS7 with LT only (Figure 3b and Supplementary Table S2).

CFs detected among patients < 5 years old in Mathbaria were diverse, with CFA/I±CS21, CS6 and CS1 (14.3% each) being the most prevalent. Among patients between 5 and 20 years old, there was only one sample where CF could be detected, which was CS6. In those of 20–60 years of age, CS6, CS5+CS6, CS17/19 (20% each), CFA/I±CS21 and CS2+CS3 (10% each) were the major CFs. In those of ≥60 years of age, only CS5+CS6 (75%) and CS3 (25%) were detected (Figure 3d and Supplementary Table S3). 

CS12 and CS20 were not detected from any of the sites. 

Serogroups: The major O serogroups detected in Chhatak were O1 (20%), O44 (16%), O55 (12%), O111 and O29 (8% each) and in Mathbaria, they were O1 (24%), O26 (12%), O6 (9%), O111, O114 and O78 (6% each). 

### 3.3. ETEC Toxin and CF Patterns over Two and Half Years in Mathbaria

**Figure 4:**
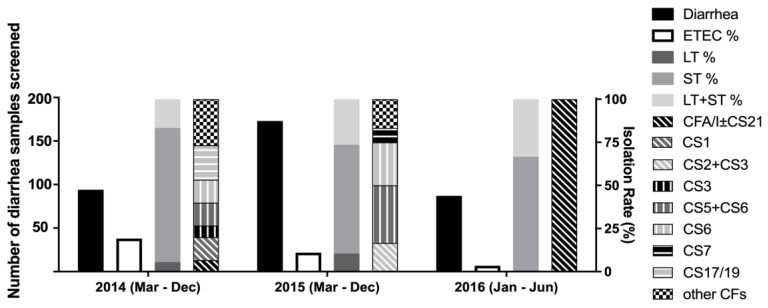
ETEC toxin gene and CF distribution over two and half years in Mathbaria. Black-filled bar: number of diarrhea samples screened, left y axis. Black line: number of ETEC cases divided by number of diarrhea samples screened, right y axis. Dark-grey-filled bar: number of LT-ETEC cases divided by total number of ETEC cases at the site, right y axis. Medium-grey-filled bar: number of ST-ETEC cases divided by total number of ETEC cases at the site, right y axis. Light-grey-filled bar: number of LT+ ST-ETEC cases divided by total number of ETEC cases at the site, right y axis. Pattern-filled bars: number of ETEC cases with specific CF(s) divided by total number of ETEC cases at the site, right y axis.

The ETEC isolation rate was higher in 2014 compared to 2015. Although the ETEC toxin distributions in the two years remained similar, the CF pattern changed from 2014 to 2015 (Figure 4). CS6 and CS5+CS6 were detected in both of the years; however, CS17/19, CS3, CS1 and CFA/I±CS21 were only noted in 2014, and CS7 and CS2+CS3 only in 2015. In 2016, from January to June, the only CF detected was CFA/I±CS21. 

### 3.4. Seasonal Occurrence of ETEC 

**Figure 5:**
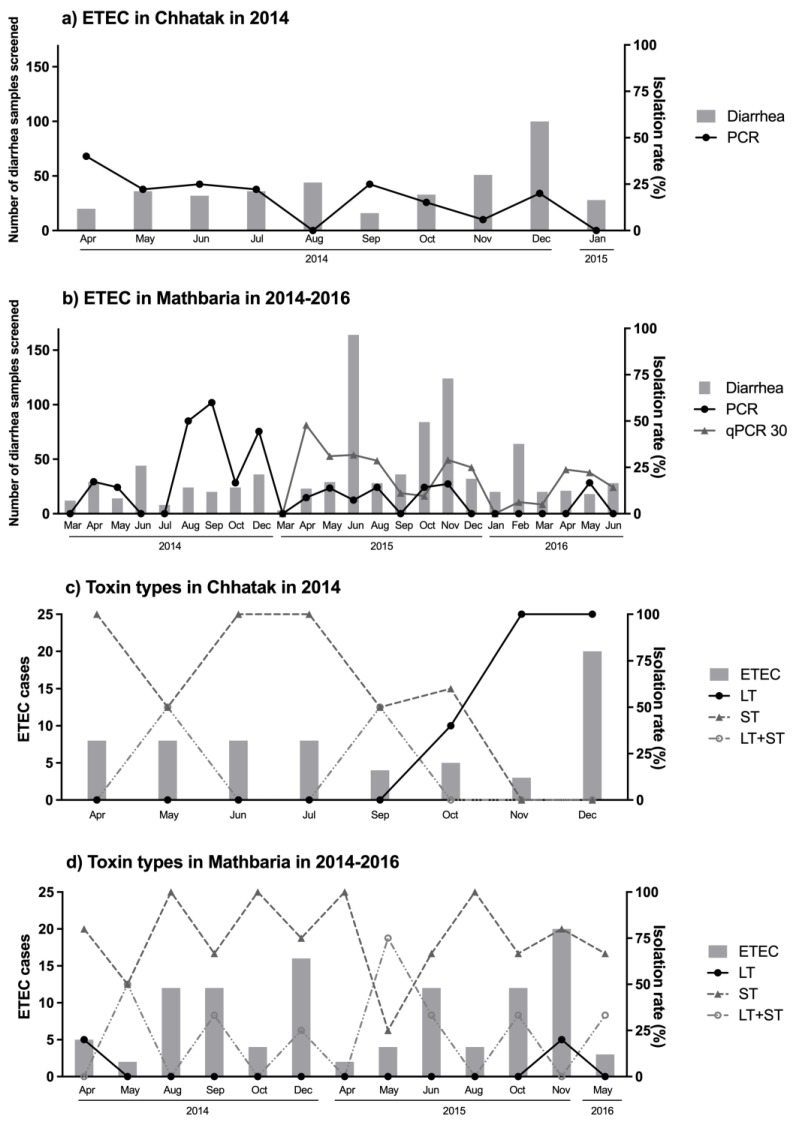
Seasonal occurrence of ETEC in Chhatak and Mathbaria. (**a**) Seasonal occurrence of ETEC in Chhatak. (**b**) Seasonal occurrence of ETEC in Mathbaria. Grey fill: number of diarrhea samples screened, left y axis. Black circle: number of ETEC cases detected by conventional PCR divided by number of diarrhea samples screened, right y axis. Grey triangle: number of ETEC cases detected by qPCR divided by number of diarrhea samples screened, right y axis. (**c**) Seasonal occurrence of ETEC toxin types in Chhatak. (**d**) Seasonal occurrence of ETEC toxin types in Mathbaria. Grey fill: number of ETEC cases, left y axis. Black circle: number of LT-ETEC cases divided by total number of ETEC cases within each month, right y axis. Grey triangle: number of ST-ETEC cases divided by total number of ETEC cases within each month, right y axis. Grey circle: number of LT-ST-ETEC cases divided by total number of ETEC cases within each month, right y axis.

ETEC was found year-round in both the sites. In Chhatak, the highest peak of ETEC (40%) was noted in April, the lowest was in November (5.9%) and it was not detected in Aug and Jan (Figure 5a). In Mathbaria, two distinct peaks were noted in the 2.5 years of surveillance, one in pre-monsoon from April to June and the second peak in the fall from August to November (Figure 5b). When ST-ETEC decreased, LT+ST-ETEC increased throughout the surveillance period in both the sites. LT-ETEC was higher in the fall and winter (Figure 5c,d).

### 3.5. Clinical Severity of ETEC Diarrhea

**Figure 6:**
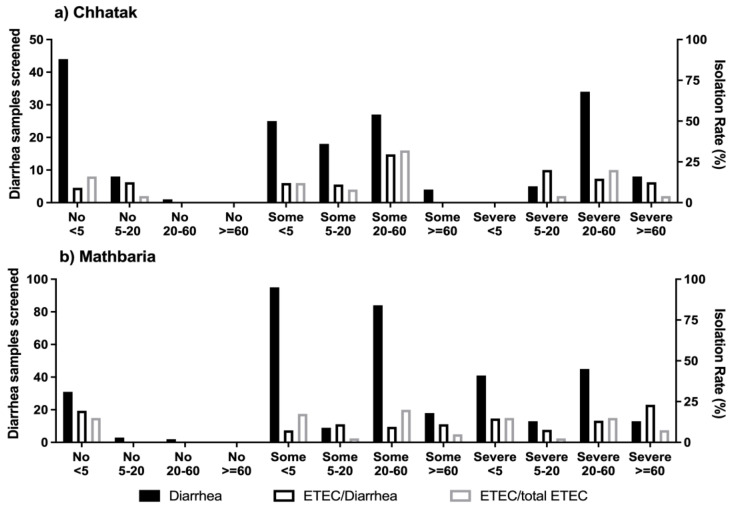
Dehydration due to ETEC diarrhea by age. Any diarrhea and ETEC-positive diarrhea cases by the severity of dehydration among the age groups in (**a**) Chhatak and (**b**) Mathbaria. Black-filled bar: number of diarrhea samples screened, left y axis. Black-outlined bar: number of ETEC cases divided by number of diarrhea samples screened, right y axis. Grey-outlined bar: number of ETEC cases divided by total number of ETEC cases, right y axis.

Among the patients with ETEC diarrhea, 35.4% (23) had severe dehydration and 47.7% (31) had some; 46.5% (20) had vomiting; 57.7% (15) had abdominal cramps; 62.8% (27) of the patients received oral rehydration solution; 4.6% (3) required IV fluid; and 74.4% (32) required hospitalization (Supplementary Table S4). The proportion of patients with ETEC diarrhea who had severe dehydration was highest among those 20–60 years old in Chhatak (71.4%) and among those < 5 and 20–60 years old in Mathbaria (37.5% each) (Figure 6). Among the samples from the patients who were ETEC-negative from *E. coli* isolates by the conventional PCR but qPCR-positive for ETEC, 36% (16 of 44) had severe dehydration, and 64% (28 of 44) had some dehydration. There was no significant difference in morbidity between the proportion of ETEC-positive and ETEC-negative diarrhea patients (Supplementary Table S4). 

**Figure 7:**
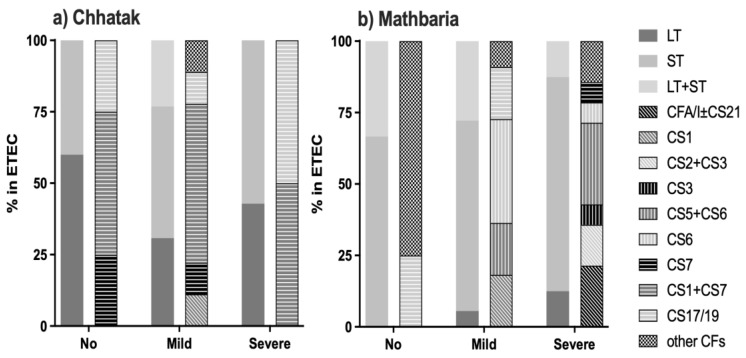
ETEC toxin and colonization factors associated with dehydration. (**a**) ETEC CFs by the severity of dehydration in Chhatak and (**b**) Mathbaria. Data are shown as the number of ETEC cases with each CF divided by the number of ETEC cases among patients with different dehydration severity.

In Chhatak, both LT-ETEC and ST-ETEC with CS17/19 (50%) and CS1+CS7 (50%) were isolated from the cases with severe dehydration; however, these CFs were also noted in the patients with mild and no dehydration (Figure 7a and Supplementary Table S5). In Mathbaria, patients positive for ST-ETEC and with CS5+CS6 (28.6%), CFA/I±CS21 (21.4%) and CS2+CS3 (14.3%) had severe dehydration (Figure 7b and Supplementary Table S5). Among the patients with no dehydration, 75% had minor CFs. 

We analyzed the ETEC diarrhea cases by the sources of water for drinking, bathing and washing utensils and clothes used by the patients. While in Chhatak, the majority of the patients with ETEC diarrhea used improved water sources like tube wells, in coastal Mathbaria, due to the high salinity of the ground water, a high proportion used unimproved sources of water like pond water. There were no significant differences in the water sources in the ETEC and non-ETEC diarrhea patients (Supplementary Figure S1). 

We estimated the proportion of ETEC diarrhea cases that could be avoided if a vaccine like the most advanced ETEC vaccine, ETVAX [8], was introduced in Chhatak and Mathbaria. ETVAX vaccine includes CFA/I, CS3, CS5 and CS6, along with LT. As described in Table 3, 59% and 76% of the ETEC diarrhea cases would be directly covered by the vaccine in Chhatak and Mathbaria, respectively. If an ETVAX-like vaccine provides cross protection from other CFs that are antigenically related to the CFs that are included in the vaccine and shown to induce immune responses in adults and children [23,24,25], 91% and 92% of the ETEC diarrhea cases in Chhatak and Mathbaria, respectively, would be protected by this vaccine (Table 3). 

## 4. Discussion

Our study in the two geographically distinct areas of Chhatak and Mathbaria in Bangladesh showed wide variations in ETEC epidemiology by location and age group. ETEC was found as a major cause of diarrhea in both the areas, and not only children less than five years of age but also adults were at risk of ETEC diarrhea with severe clinical outcomes that required urgent care at the hospitals. 

We showed that within a country with a high burden of diarrheal disease, there were considerable variations in the ETEC burden and the types of circulating ETEC strains. In 2014, while the rate of patients with ETEC-positive diarrhea seeking care at the hospitals was 14% in the north in Chhatak, the rate was higher (19%) in the south in coastal Mathbaria. These rates were higher than those which were reported from the central part of Bangladesh, which were 8–11% in urban Dhaka and 3% in rural Mizapur in 2010–11 [9,26]. The ETEC diarrhea rate and the prevalence of CF types varied over the years in Mathbaria, while the distributions of the toxin types remained unchanged. With qPCR, the isolation rate of ETEC was doubled compared to conventional PCR. 

The two seasonal peaks of ETEC in pre-monsoon and in fall in Mathbaria were similar to those reported by Dhaka [13], while in Chhatak, ETEC was more or less found with similar frequencies all year round, except in August, October and January, when no ETEC cases were isolated. Interestingly, there are seasonal changes in the prevalence of the toxin types of ETEC, which underscores the importance of longitudinal surveillance studies. 

An important finding in this study is the high isolation rates of ETEC in the adults aged 20–60 years who required care at the hospitals. In Chhatak, while the proportion of ETEC diarrhea was highest among the age group of 20–60 years, which was ~2-fold higher than patients under 5 years old, in Mathbaria, the ETEC proportion was highest and similar in both <5-year-old and between 20- and 60-year-old patients. The proportion of ETEC diarrhea was much lower in the older children to younger adults, which may suggest immunity from multiple previous ETEC infections. In this age group, the proportion of LT-ETEC was very low in Chhatak and nonexistent in Mathbaria, even with qPCR, and while in Chhatak, the CFs detected were CS17/19 and CS1, in Mathbaria there was only CS6. It should be noted that LT is highly immunogenic, while ST and CS6 are not, and therefore, it is likely that this age group is protected by immunity from previous infections with the ETEC types that are immunogenic. Notably, ETEC was isolated from a considerable number of patients over 60 years of age in Mathbaria. A higher proportion of ETEC diarrhea in patients > 5 years old was also reported from the central region of Bangladesh [9,27], although the age group that is at the highest risk among the adults was not identified. 

A notable finding in this study is the variations in the types of ETEC, the toxins and the colonization factors in the two study areas. While in Chhatak, the majority of the circulating ETEC strains were LT-ETEC (40%) and ST-ETEC (48%), in Mathbaria, there were very few LT-ETEC (7.5%) cases in the 2.5 years of surveillance. Even using qPCR detection, the proportion of LT-ETEC cases remained low. The ETEC toxin profiles in Dhaka were different from these two study areas, with 43% LT+ST-ETEC, followed by 30% ST-ETEC and 27% LT-ETEC [28]. The distribution of the CFs was distinctly different between the north and south of Bangladesh. In Chhatak, the major CFs were CS1+CS7, CS17/19, CS7 and CS1, while in Mathbaria CS5+CS6, CS6 and CFA/I+CS21 were the most prevalent. The types of ETEC CFs reported from Dhaka were a mix between the CFs found from the north and south in this study. The major CFs (≥10%) in Dhaka were CFAI±CS21, CS5+CS6, CS7, CS17, CS6 and CS14 [28]. Toxins and CFs (CFA/I, CS1-6 and LTB) are the major antigens in the current ETEC vaccine candidates, and therefore, the distributions of the toxin types and CFs at the subnational level are crucial to determine the potential effectiveness of the ETEC candidate vaccines. The primary CFs found in Chhatak, CS17/19 and CS7, are not included in either ACE527 [28] or the most advanced ETEC vaccine candidate, ETVAX [29], although ETVAX ETEC vaccine has been shown to induce responses to CS17 and CS7 in Swedish adults and Bangladeshi and Zambian children [23,24,25]. Nevertheless, in our analysis, approximately half of the ETEC diarrhea cases in Chhatak and three fourths in Mathbaria could be avoided if an ETVAX-like ETEC vaccine is introduced. We also found a considerable variation in toxins and CF types by the age of patient. Less heterogeneity of CFs was noted among the older children–young adult group. 

In this study, about half of the ETEC diarrhea patients presented with severe or some dehydration, vomiting and abdominal cramps. Interestingly, many of the ETEC diarrhea patients with severe dehydration were between 20 and 60 years old in Chhatak and <5 years old in Mathbaria. Notably, qPCR detected an additional sixteen ETEC-positive diarrhea patients who had severe dehydration, who were missed by the isolate-based conventional PCR. 

This study has several strengths, which include longitudinal hospital-based surveillance for multiple years among patients from all ages, inclusion of two geographically distinct areas in a country, and comparing isolation rates by culture and qPCR and variations in the distributions of toxins and CFs by location and age group. This study also has limitations; we tested fourteen CFs, which, although they included all the major CFs except CS14, may have missed some minor CFs which are circulating in these areas. We were not able to determine the seasonality in more than one year in Chhatak, and we could only perform qPCR with stool samples in Mathbaria. Since the toxin and CF distributions remained similar over the years, these data may reflect the current epidemiology of ETEC in these areas. We were not able to estimate ETEC prevalence in individuals without diarrhea to calculate the attributable fractions of ETEC in the diarrhea cases. 

Conclusions: This study underscores the importance of capturing subnational, seasonal and age-specific variations in ETEC epidemiology. This study has revealed that in addition to children < 5 years old, adults at 20–60 years of age are also at high risk of getting severe ETEC diarrhea requiring hospitalization. Therefore, vaccination against ETEC should also consider targeting adults. 

Disclaimer: The content of this article is solely the responsibility of the authors and does not necessarily represent the official views of the NIH/NIAID. None of the authors have any conflict of interest.

## Figures and Tables

**Table 1:** ETEC prevalence in Chhatak and Mathbaria over the years.

	Chhatak	Mathbaria			
	April 2014–January 2015	March 2014–June 2016	March–December 2014	March–December 2015	January–June 2016
Total diarrhea patients screened	174	354	94	173	87
Male–Female Ratio	0.71	1.3	1.09	1.75	0.89
Patients with ETEC	25 (14.4%)	40 (11.3%)	18 (19.1%)	19 (11.0%)	3 (3.4%)
Male–Female Ratio for ETEC	1.08	0.82	0.8	0.9	0.5

**Table 2:** ETEC toxin types at the study sites in each year of surveillance.

	PCR from *E. coli* Isolates n (%)				qPCR from Stool n (%)			
	Total ETEC	LT-ETEC	ST-ETEC	LT+ST-ETEC	Total ETEC	LT-ETEC	ST-ETEC	LT+ST-ETEC
Chhatak 2014–2015	25	10 (40.0%)	12 (48.0%)	3 (12.0%)	ND			
Mathbaria 2014–2016	40	3 (7.5%)	28 (70.0%)	9 (22.5%)				
Mathbaria 2014	18	1 (5.6%)	14 (77.8%)	3 (16.7%)	ND			
Mathbaria 2015	19	2 (10.5%)	12 (63.2%)	5 (26.3%)	50	10 (20.0%)	22 (44.0%)	18 (36%)
Mathbaria 2016	3	0	2 (66.7%)	1 (33.3%)	12	4 (33.3%)	2 (16.7%)	6 (50.0%)

**Table 3:** Estimated protection from ETEC diarrhea cases from ETVAX-like ETEC vaccine in Chhatak and Mathbaria.

ETEC Strain	Proportion of ETEC Strains that Will Be Covered	
	Chhatak	Mathbaria
LT-ETEC	40%	7.5%
LT-ETEC + LT+ST-ETEC	52%	30%
LT-ETEC + LT+ST-ETEC + CFs (CFA/I, CS3, CS5 and CS6)	59%	76%

## Data Availability

Data are contained within the article and supplementary materials.

## Supplementary Materials

The following supporting information can be downloaded at https://www.mdpi.com/article/10.3390/microorganisms12020359/s1: Supplementary Table S1: Primers for quantitative PCR (qPCR) and conventional PCR; Supplementary Table S2: Association of toxins and major CFs in ETEC isolates; Supplementary Table S3: Association of age groups and major CFs in ETEC isolates; Supplementary Table S4: Clinical severity of ETEC and Non-ETEC diarrhea; Supplementary Table S5: Association of dehydration and major CFs in ETEC isolates; Figure S1: Water sources for drinking, bathing and washing among ETEC patients. References [21,30] are cited in the supplementary materials.
