# Supplementary material for: Epidemiology of Enterotoxigenic Escherichia coli among Children and Adults Seeking Care at Hospitals in Two Geographically Distinct Rural Areas in Bangladesh
Source: Microorganisms. 2024 Feb 9;12(2):359. doi: 10.3390/microorganisms12020359 (PMC10891752; doi:10.3390/microorganisms12020359)
Supplement: Supplementary file 1 [file microorganisms-12-00359-s001.zip › microorganisms-2812450-supplementary.pdf]

Supplemental Data

Supplement Table S1. Primers for quantitative PCR (qPCR) and conventional PCR

qPCR primers			
**Enterotoxin**	**Primer**	**Primer sequence 5′– 3′**	**Reference**
STh	STh-rtF1	AAGTGGTCCTGAAAGCATGAATAGTAG	1
	STh-rtR1	ACCCGGTACAAGCAGGATTACA	
STp	STp-rtF1	GCAAAATCCGTTTAACTAATCTCAAA	1
	STp-rtR1	AATTGCCAACATTAGCTTTTTCATG	
LT	LT-RTf	GGCAGGCAAAAGAGAAATGG	1
	LT-RTr	TCCTTCATCCTTTCAATGGCT	
Conventional PCR Primers			
STh	SThF	TTCACCTTTCCCTCAGGATG	2
	SThR	CTATTCATGCTTTCAGGACCA	
STp	STpF	TCTTTCCCCTCTTTTAGTCAG	2
	STpR	ACAGGCAGGATTACAACAAAG	
LT	LTF	ACGGCGTTACTATCCTCTC	2
	LTF	TGGTCTCGGTCAGATATGTG	

Supplemental Table S2. Association of toxins and major CFs in ETEC isolates

Toxin gene present	CF type(s) produced	No. (%) of isolates
Chhatak		
ST	CS1+CS7	4 (50)
	CS17/19	3 (37.5)
	Other CFs	1 (12.5)
LT and ST	CS1	1 (50)
	CS1+CS7	1 (50)
LT	CS7	2 (40)
	CS1+CS7	3 (60)
Mathbaria		
ST	CFA/I+CS21	2 (9.1)
	CS1	2 (9.1)
	CS2+CS3	1 (4.5)
	CS3	1 (4.5)
	CS5+CS6	5 (22.7)
	CS6	5 (22.7)
	CS17/19	2 (9.1)
	Other CFs	4 (18.2)
LT and ST	CS2+CS3	1 (20)
	CS5+CS6	1 (20)
	CS17/19	1 (20)
	Other CFs	2 (40)
LT	CFA/I+CS21	1 (50)

Supplemental Table S3. Association of age groups and major CFs in ETEC isolates

Age groups	CF type(s) produced	No. (%) of isolates
Chhatak		
<5	CS7	2 (33.3)
	CS1+CS7	3 (50)
	CS17/19	1 (16.7)
5-20	CS1	1 (33.3)
	CS17/19	2 (66.7)
20-60	CS1+CS7	5 (83.3)
	Other CFs	1 (16.7)
Mathbaria		
<5	CFA/I+CS21	2 (14.3)
	CS1	2 (14.3)
	CS2+CS3	1 (7.1)
	CS5+CS6	1 (7.1)
	CS6	2 (14.3)
	CS7	1 (7.1)
	CS17/19	1 (7.1)
	Other CFs	4 (28.6)
5-20	CS6	1 (100)
20-60	CFA/I+CS21	1 (10)
	CS2+CS3	1 (10)
	CS5+CS6	2 (20)
	CS6	2 (20)
	CS17/19	2 (20)
	Other CFs	2 (20)
>=60	CS3	1 (25)
	CS5+CS6	3 (75)

Supplement Table S4. Clinical severity of ETEC and Non ETEC diarrhea.

	Non ETEC diarrhea	ETEC diarrhea	p value
	(N=463)	(N=65)	
Dehydration Status			0.582
No	78 (16.8%)	11 (16.9%)	
Some	249 (53.8%)	31 (47.7%)	
Severe	136 (29.4%)	23 (35.4%)	
Vomiting			0.947
No	116 (51.6%)	23 (53.5%)	
Yes	109 (48.4%)	20 (46.5%)	
Fever			0.671
No	215 (95.6%)	42 (97.7%)	
Yes	6 (2.7%)	1 (2.3%)	
Abdominal cramp			0.751
No	61 (48.0%)	11 (42.3%)	
Yes	66 (52.0%)	15 (57.7%)	
IV fluid			0.197
No	414 (89.4%)	62 (95.4%)	
Yes	49 (10.6%)	3 (4.6%)	
ORT			0.505
No	69 (30.7%)	16 (37.2%)	
Yes	156 (69.3%)	27 (62.8%)	
Hospitalization			0.413
No	75 (33.3%)	11 (25.6%)	
Yes	150 (66.7%)	32 (74.4%)	

Data for all the variables from all the patients were not available

Supplement Table S5. Association of dehydration and major CFs in ETEC isolates

Dehydration	CF type(s) produced	No. (%) of isolates
Chhatak		
No	CS7	1 (25)
	CS1+CS7	2 (50)
	CS17/19	1 (25)
Mild	CS1	1 (11.1)
	CS7	1 (11.1)
	CS1+ CS7	5 (55.6)
	CS17/19	1 (11.1)
	Other CFs	1 (11.1)
Severe	CS1+CS7	1 (50)
	CS17/19	1 (50)
Mathbaria		
No	CS17/19	1 (25)
	Other CFs	3 (75)
Mild	CS1	2 (18.2)
	CS5+CS6	2 (18.2)
	CS6	4 (36.4)
	CS17/19	2 (18.2)
	Other CFs	1 (9.1)
Severe	CFA/I+CS21	3 (21.4)
	CS2+CS3	2 (14.3)
	CS3	1 (7.1)
	CS5+CS6	4 (28.6)
	CS6	1 (7.1)
	CS7	1 (7.1)
	Other CFs	2 (14.3)

Drinking water sources of the patients with ETEC diarrhea: Among the ETEC positive patients, in Chhatak, 100% (25/25) used improved drinking (tube well), 56% (14 /25) improved bathing and washing water sources. In Mathbaria, 30% (12/40) used improved drinking water sources and only 7.5% used improved bathing and water sources. Pond water was used by 30% (10/40) of the ETEC diarrhea patients. There were no significant differences in the water sources in the ETEC and non-ETEC diarrhea patients.

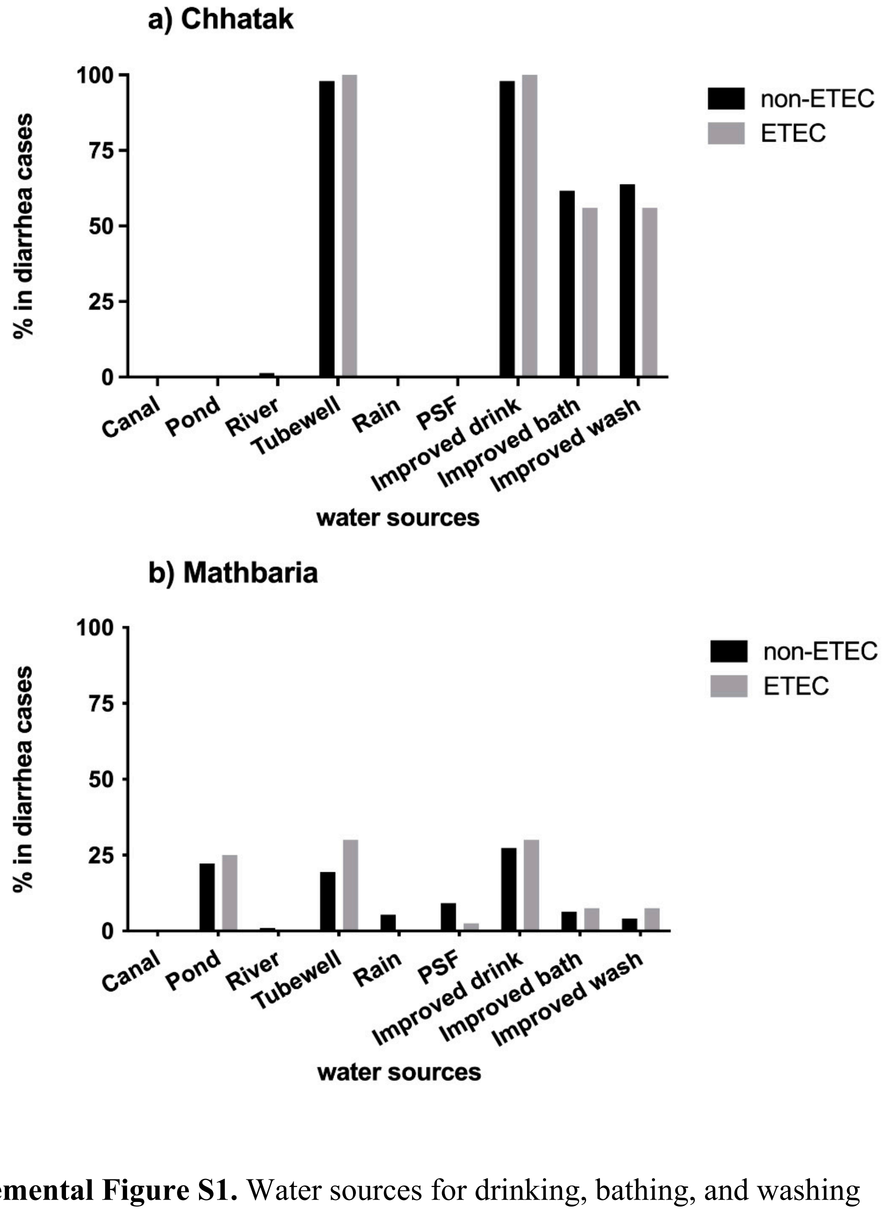

Supplemental Figure S1. Water sources for drinking, bathing, and washing among the ETEC patients. (**a**) Chhatak. (**b**) Mathbaria. Black bar: number of

non-ETEC diarrhea cases using each water source divided by the total number of non-ETEC diarrhea cases at the site. Grey bar: number of ETEC diarrhea cases using each water source divided by the total number of ETEC diarrhea cases at the site.

References

1. Lothigius, Å.; Janzon A.; Begum, Y.; Sjöling, Å.; Qadri, F.; Svennerholm, A.-M., Bölin, I. Enterotoxigenic *Escherichia coli* is detectable in water samples from an endemic area by real-time PCR, *Journal of Applied Microbiology*, **2008**, 104(4): 1128–1136
2. Rodas, C.; Iniguez, V.; Qadri, F.; Wiklund, G.; Svennerholm, A.M'; Sjöling, A. Development of multiplex PCR assays for detection of enterotoxigenic Escherichia coli colonization factors and toxins. *J Clin Microbiol* **2009,** 47(4):1218-20
